# Automated Red Cell Exchange in the Management of Sickle Cell Disease

**DOI:** 10.3390/jcm10040767

**Published:** 2021-02-15

**Authors:** Dimitris A. Tsitsikas, Saket Badle, Rhys Hall, John Meenan, Oloruntoyin Bello-Sanyaolu, Funmilayo Orebayo, Jibril Abukar, Mohamed Elmi, Afsana Mulla, Shalini Dave, Natasha Lewis, Manisha Sharma, Basabi Chatterjee, Roger J. Amos

**Affiliations:** 1 Haemoglobinopathy Service, Department of Haematology, Homerton University Hospital NHS Foundation Trust, London E9 6SR, UK; saket.badle1@nhs.net (S.B.); rhys.hall@nhs.net (R.H.); johnmeenan@nhs.net (J.M.); oloruntoyin.bello-sanyaolu@nhs.net (O.B.-S.); funmilayo.ola-adenekan@nhs.net (F.O.); j.abukar@nhs.net (J.A.); mohamed.elmi2@nhs.net (M.E.); afsana.mulla@nhs.net (A.M.); minal.dave3@nhs.net (S.D.); Natasha.Lewis@homerton.nhs.uk (N.L.); basabi.chatterjee@nhs.net (B.C.); roger.amos52@googlemail.com (R.J.A.); 2 Department of Biochemistry, Homerton University Hospital NHS Foundation Trust, London E9 6SR, UK; manisha.sharma2@nhs.net

**Keywords:** sickle cell disease, automated red cell exchange, alloimmunisation, iron overload

## Abstract

Red cell transfusion represents one of the cornerstones of the chronic management of sickle cell disease, as well as its acute complications. Automated red cell exchange can rapidly lower the number of circulating sickle erythrocytes, without causing iron overload. Here, we describe our experience, having offered this intervention since 2011. A transient reduction in the platelet count by 61% was observed after the procedure. This was not associated with any haemorrhagic complications. Despite exposure to large volumes of blood, the alloimmunisation rate was only 0.027/100 units of red cells. The absence of any iron loading was confirmed by serial Ferriscans, performed over a number of years. However, patients with advanced chronic kidney disease showed evidence of iron loading due to reduced innate haemopoiesis and were subsequently switched to simple transfusions. A total of 59% of patients were on regular automated red cell exchange with a history of recurrent painful crises. A total of 77% responded clinically, as evidenced by at least a 25% reduction in their emergency hospital attendance for pain management. The clinical response was gradual and increased the longer patients stayed on the program. The earliest sign of clinical response was a reduction in the length of stay when these patients were hospitalised, indicating that a reduction in the severity of crises precedes the reduction in their frequency. Automated red cell exchange also appeared to be beneficial for patients with recurrent leg ulcers and severe, drug resistant stuttering priapism, while patients with pulmonary hypertension showed a dramatic improvement in their symptoms as well as echocardiographic parameters.

## 1. Introduction

Red cell transfusion improves tissue oxygen delivery and lowers the proportion of sickle erythrocytes in the circulation, making it a key intervention in both the chronic management of sickle cell disease (SCD) as well as in its acute complications. Even though historically chronic transfusion therapy has been used mainly for primary and secondary prevention of cerebrovascular events, it is increasingly considered for other indications, such as history of acute chest syndrome (ACS) and recurrent painful crises (RPC) [1,2].

Simple transfusion is easy to deliver and does not require specialist staff or equipment but is associated with iron overload and is less effective in reducing the Hb S level. Exchange transfusion can be manual when the patient’s blood is removed and replaced by donor red cells and replacement fluid, usually normal saline to maintain isovolaemia. This process is laborious, less effective in reducing the Hb S level, especially in the acute situation and can still lead to a degree of iron overload. Automated red cell exchange (a-RCE) is performed through an apheresis system where the patient’s own red cells are removed and replaced with donor red cells before the blood is returned to the patient. This process requires specialist staff and equipment but can rapidly reduce the Hb S level, avoiding iron loading, hence it is the recommended mode of delivering chronic transfusions for patients with SCD [3].

In this article we describe our experience at the sickle cell and thalassaemia Centre at Homerton Hospital, London, UK, providing regular a-RCE as a disease-modifying intervention in our institution since June 2011 and present data on several different aspects of the procedure.

## 2. Methodology

### 2.1. Definitions

Determining how long individual patients have been on the program, the number of years was rounded to the closest whole number; e.g., a patient receiving treatment for 1.6 years is classified as “2 years” while a patient receiving treatment for 1.4 years as “1 year”. Patients who had interruptions in their treatment of less than 6 months appear as one episode while 5 patients who had interruptions of longer than 6 months are reported as separate episodes.

Iron overload was defined as mild, if the liver iron concentration (LIC) by liver MRI (Ferriscan) was 2–6.9, moderate for LIC 7–14.9 and severe for LIC ≥ 15 mg/g of dry weight.

Sickle cell nephropathy (SCN) was defined as persistent proteinuria or evidence of chronic kidney disease (CKD); CKD was defined as an estimated glomerular filtration rate (eGFR) of less than 90 mL/min [4]. Patients are reported as suffering of pulmonary hypertension (PH) only if the diagnosis is confirmed by right heart catheterisation (RHC). However, in our analysis, we also include symptomatic patients with a TRVmax ≥ 2.8 m/s by echocardiography (PH?).

Response to treatment for patients with leg ulcers (LU) was based on objective evidence of recurrence, as well as patient reported improvement, while response for stuttering priapism (SP) was solely based on patient reported improvement. For patients on a-RCE for RPC, assessment of response was conducted by comparing the rate of hospital attendance for pain management after commencing a-RCE to the year prior to entering the programme. A reduction of ≥25% was required to classify patients as “responding” to treatment.

### 2.2. Patients

**Figure 1:**
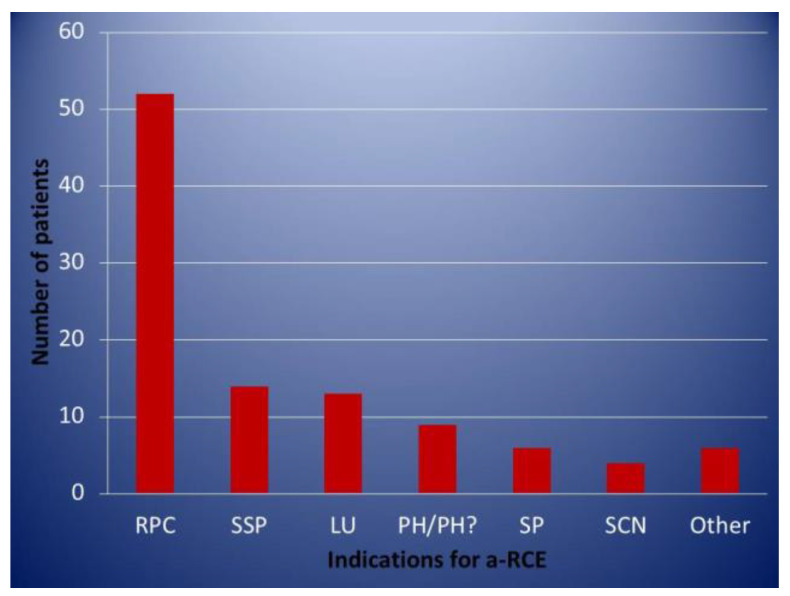
Indications for regular automated red cell exchange. RP = recurrent painful crises, SSP = secondary stroke prevention, LU = leg ulcers, PH = pulmonary hypertension, SP = stuttering priapism, SCN = sickle cell nephropathy.

**Figure 2:**
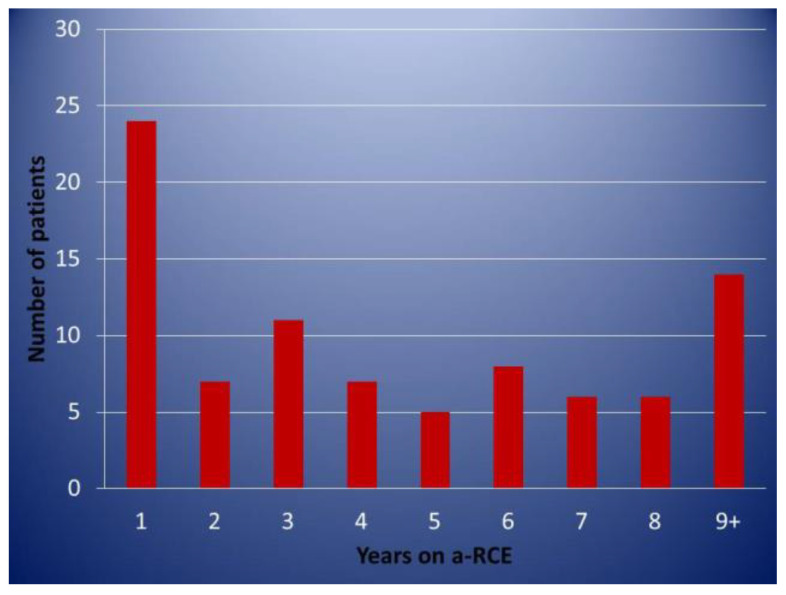
Distribution of patients according to duration (years) of continuous automated red cell exchange.

Patients on an elective regular programme were only included in this analysis if they had completed at least 1 year of treatment. A total of 88 patients fitting the above criteria were identified. Data were retrieved from the electronic patient record (EPR) as well as the patients’ individual transfusion protocols. A total of 46 patients were female and 42 male, with ages ranging from 18–68 (median age 37). A total of 69 (78%) patients had Hb SS/Sβ^0^, 17 (19%) Hb SC, 2 (2%) Hb Sβ^+^, and 1 (1%) patient had Hb SD^Punjab^. Of these, 68 remained on the programme, while 20 were discontinued for different reasons. A total of 52 (59%) patients entered the programme for management of recurrent painful crises (RPC). All these patients were first considered for treatment with hydroxycarbamide and had either declined or were intolerant/had contraindications. The indications for regular a–RCE can be seen in Figure 1. A total of 19 (22%) patients had more than one indication. The length of time individual patients were on the programme is shown in Figure 2.

### 2.3. The Procedure

The Spectra Optia apheresis system (Terumo BCT) was used by nurses trained and signed as being competent at performing the procedure. Red cell units meeting specifications as per national guidelines for patients with SCD [5] were provided by the National Blood Service. All elective procedures were performed as day cases (ambulatory a-RCE) in our day unit with same day discharge for the vast majority of patients. Even though when we first started, intravenous access was via temporary femoral central catheters (Vas-Cath) for the majority of patients, progressively, an increasing number have the procedure using peripheral access. Currently, 48 of the 68 patients (71%) have the procedure performed through peripheral cannulas, 18 G, 20 G or 22 G according to body habitus, while an additional 15 (22%) through peripheral deep access lines 18 or 20 G, inserted under ultrasound guidance by trained nurses in our day unit.

The infusion rate of the citrate anticoagulant is set at 0.6 mL/min when patients first enter the programme and then, if tolerated, increased to 0.8 mL/min.

The red cell volume, required peri-procedure investigations and targets for the haematocrit (Hct) and Hb S/S&C are specified in each patient’s individual transfusion protocol. During the study period, the mean number of red cell units used per procedure was 8.3, with a mean interval between procedures of 6.7 weeks.

### 2.4. Monitoring

The target pre-transfusion Hb S or Hb S&C level was set at 30% for patients treated for secondary stroke prevention (SSP) and lower (20–30%) for patients with pulmonary hypertension. Even though the target was initially set at 30% for patients with RPC, higher sickle percentages were acceptable if there was evidence of clinical response. The target post-transfusion Hct was set between 29 and 32, depending on the patient’s baseline.

Iron status assessment included serum ferritin measurements, with each transfusion and liver MRI (Ferriscan) being conducted yearly or every 2 years for patients with or without known iron overload, respectively, as per departmental guidelines. Patients whose serum ferritin was consistently below 500 mcg/L were not offered imaging.

Equally, specific assessments, such as echocardiography and brain magnetic resonance imaging/angiography (MRI/MRA), were performed for suitable patients to assess the efficacy of the intervention. All transfusion protocols contain information about transfusion requirements, iron assessments and iron chelation, if applicable, in addition to alloimmunisation status, as well as virology screening tests. Each of these is reviewed at least yearly at the haemoglobinopathy multidisciplinary team (MDT) meeting (Supplemental File 1).

### 2.5. Statistical Considerations

A limited statistical analysis has been carried out as this is a retrospective study. Graphpad Prism version 9 software was used and statistical tests have been specified where relevant.

### 2.6. Limitations

This is a retrospective analysis of our experience offering a-RCE as a standard of care over a period spanning just under 10 years. Data on some occasions may be incomplete or missing and subject to appropriate documentation and coding. When assessing the response to treatment, other concurrent interventions for individual patients were not taken into consideration; however, as this population was treated in the same service, we do not expect significant discrepancies.

## 3. Results and Discussion

### 3.1. Haematological Parameters

A total of 3107 elective procedures using 25,649 units of red cells were performed from June 2011 to October 2020. The pre-and post-procedure haematological parameters achieved are summarised in Table 1. The mean pre a-RCE Hb S/S&C level was 44%. This is largely explained by the fact that a large number of patients on the programme for RPC achieved clinical responses with sickle levels greater than 30%. The other contributing factor was patients’ decision to avoid a more “intense” programme. Extremely high values reflect patient’s starting a-RCE for the first time or procedures that had to be terminated earlier than planned.

Having previously analysed a wide range of haematological and biochemical parameters and found no significant changes other than for platelet count [6], we have not looked into these in the current analysis.

Significant post-procedural thrombocytopenia was confirmed with a mean 61% reduction in the platelet count (*p* value < 0.0001, Wilcoxon *t* test, Graphpad Prism v 9). No patients had any significant bleeding complications and platelet recovery was rapid, with platelets returning to pre-transfusion levels within a few days. However, this effect can be clinically significant if the patient is on antiplatelet agents or anticoagulants or when a-RCE is performed shortly before surgical procedures.

### 3.2. Alloimmunisation

A total of seven patients were known to have alloantibodies before commencing a-RCE; none of these patients developed any new alloantibodies. Six of the remaining 81 (7.5%) patients on regular a-RCE developed a total of seven new alloantibodies: two anti Kp(a), two anti-E, one anti-e, one anti-C and one anti-Cw. This gave an alloimmunisation rate of 0.027/100 units of red cells. Examining all the transfusion records of the patients who developed anti-Rh, none of them had received any red cell units positive for the relevant antigens in our hospital. “Unexplained” anti-Rh formation has previously been reported and attributed to the presence of variant Rh alleles that cannot be distinguished with routine serologic typing [7]. All six patients continued with regular a-RCE, receiving appropriate red cell units. One additional patient developed an auto anti-e. There were no haemolytic transfusion reactions reported in this group of patients. However, there were three episodes of post transfusion hyper haemolysis syndrome, a severe delayed haemolytic transfusion reaction leading to destruction of both donor and recipient red cells resulting in a drop in Hb to lower than pretransfusion levels [8], after one-off procedures (one emergency for ACS and two electives prior to surgery).

A low rate of alloimmunisation in patients receiving regularly large volumes of red cells has previously been reported and it has been hypothesised that it may be the result of the immune system being “overwhelmed” [9] or that the low rate of alloantibody formation is the result of suppression of the chronic inflammatory state characteristic of SCD in patients receiving regular transfusions [10]. We have, indeed, previously reported a very low rate of alloimmunisation [6] and that documented here is even lower, which may be explained by the longer period that patients have been on regular a-RCE, in keeping with the latter theory.

### 3.3. Iron Loading

**Figure 3:**
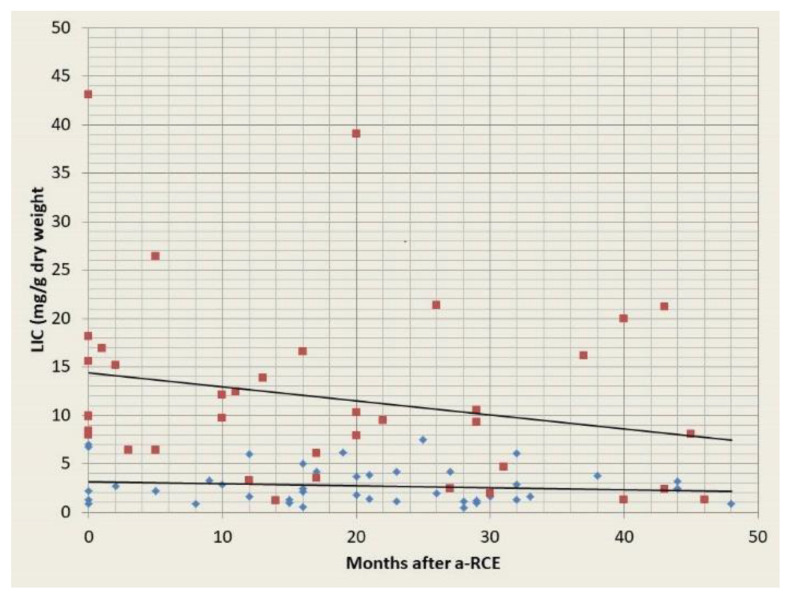
Iron assessment by serial Ferriscans for patients with moderate/severe (red squares) and mild or no pre-existing iron overload (blue diamonds). LIC = liver iron concentration, a-RCE = automated red cell exchange.

A total of 28 patients received a total of 80 serial (≥2) assessments by Ferriscan and for at least a period of 4 years. Patients with no or mild iron overload showed no evidence of iron accumulation, while patients with moderate or severe iron overload showed a clear downward trend (Figure 3). In addition, eight patients had serial MRIs but subsequently had no more for a mean period of 3 years (2–4), as the mean annual ferritin was consistently below 500 mcg/L, while 14 patients had no further Ferriscans after the first one for a mean period of 3.5 years (2–6), for the same reason. Finally, 16 patients never had a Ferriscan, as their mean ferritin level was below 500 mcg/L and remained at that level for a mean period of 4.4 years (2–8). Four patients with moderate and two patients with severe iron overload receiving iron chelation before entering the a-RCE programme have since discontinued treatment with chelating agents, after a mean 1.8 years (1–2.5). Four patients with established CKD started showing evidence of iron accumulation in the form of rising levels of serum ferritin and increased liver iron concentrations, determined by MRI, as their eGFR declined. These patients were subsequently switched to simple transfusions. Our data confirm that a-RCE is associated with no iron accumulation, with firm evidence provided from serial imaging of the liver. However, in the future we may see a degree of iron accumulation if we start using higher target haematocrit in attempt to avoid post transfusion fatigue (see Section 3.12) [11]. In exception to this, are patients with advanced CKD. Due to greatly reduced innate haemopoiesis, these patients drop their haemoglobin significantly between cycles, without a significant increase in their Hb S level, and receive more red cells than are removed. Thus, these patients are better served by simple transfusions.

### 3.4. Recurrent Painful Crises

A total of 52 patients have been on a regular programme for management of RPCs. Of these, the response of nine patients could not be assessed, as their primary site of care was a different institution. Of the remaining 43, 10 patients (23%) showed no evidence of clinical response. A total of 33 patients responded clinically, showing at least a 25% reduction in their hospital attendance for pain management. Five patients had two episodes each, as they had interruptions in their transfusions for longer than 6 months. There were no significant differences in the mean pre-transfusion Hb S levels achieved between the two groups; 42 and 36% for responding and non-responding patients respectively.

**Figure 4:**
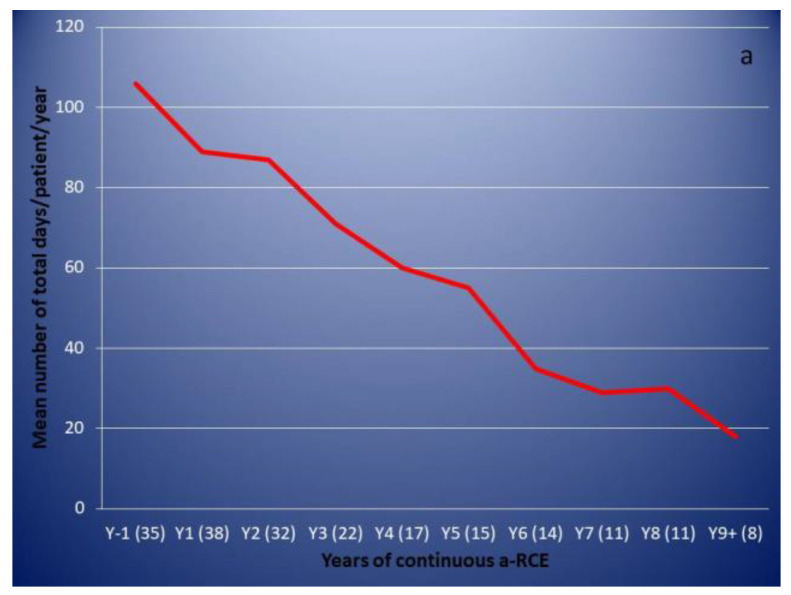

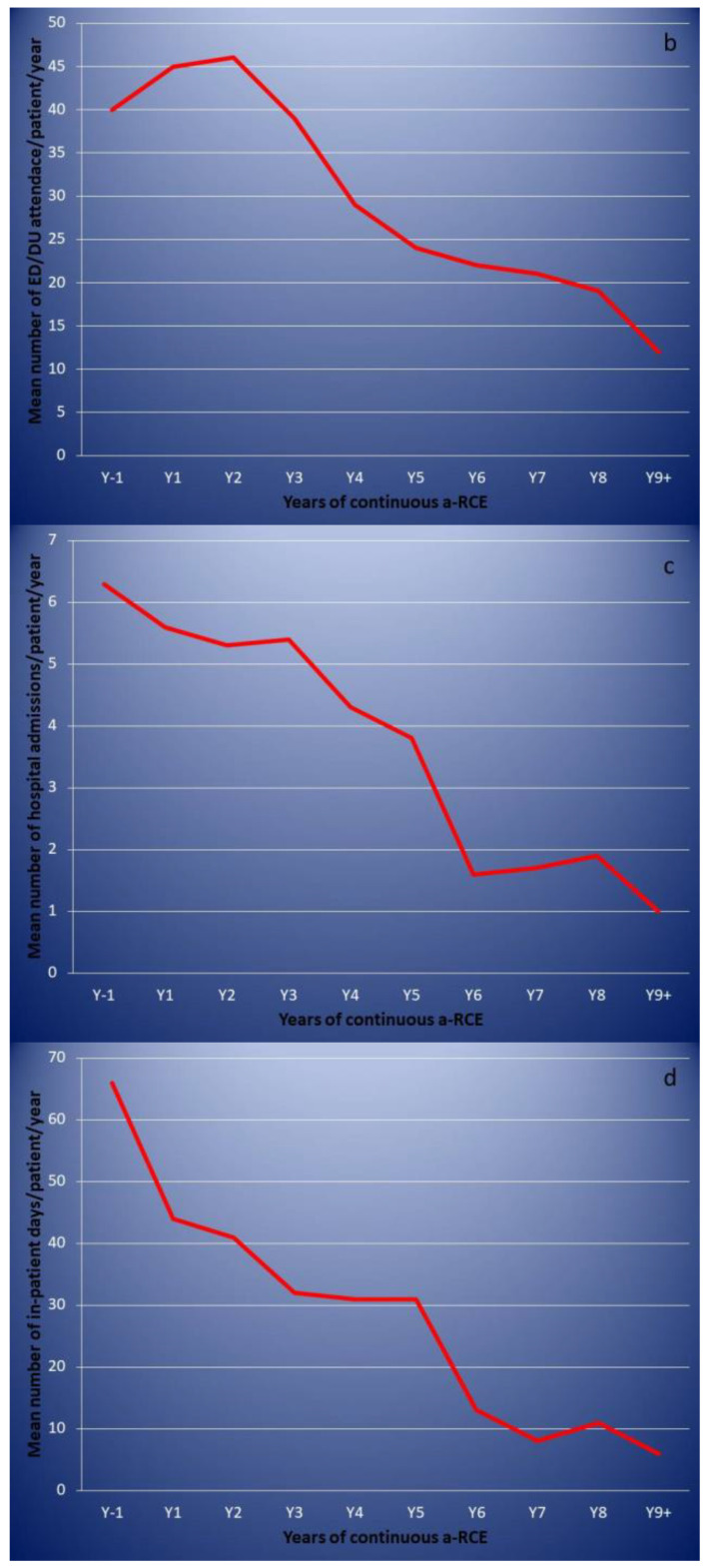
Emergency hospital attendance for pain management before and after commencing automated red cell exchange (responding patients only). Figures in brackets indicate number of episodes. (**a**): mean/patient total number of days of hospital attendance for pain managemen. (**b**): mean/patient visits to the emergency department (ED) or the haematology day unit (DU) for treatment with same day discharge. (**c**): mean/patient number of hospital admissions. (**d**): mean/patient total number of days of hospitalisation.

Responding patients showed a gradual and progressive reduction in the total number of days they had to attend hospital for pain management over the years (Figure 4a). Different patterns of responses were observed when breaking down attendance into visits to the emergency department (ED) or the haematology day unit (DU) for treatment with same day discharge (Figure 4b), number of hospital admissions (Figure 4c) and total number of days of hospitalisation (Figure 4d).

As previously observed [6,12], the response is gradual and builds over time. Responses can be slow and patients who go on to show a very clear clinical benefit may not do so until they have been on a regular programme for 2 years or more. The earliest and most pronounced sign of response is a reduction in the length of stay when patients are hospitalised, indicating that patients experience a faster and more significant improvement in the severity of their crises, rather than their frequency. For responding patients, the mean total number of days of hospital attendance/patient reduced from 106 the year before entering the programme, to 89 after the first year (16% reduction) and 87 after the second year (18% reduction). An even more modest reduction in the number of hospital admissions of 11% after the first year and 16% after the second was observed, while ED/DU attendances with same day discharge increased. However, the days of hospitalisation reduced by 33 and 38% after the first and second year, respectively. This observed difference between same day discharges and hospitalization was not statistically significant after the first year (*p* value 0.08 Fisher exact test; Graphpad Prism v 9) but showed statistical significance after the second year (*p* value 0.0419) and remained significant after the ninth year (*p* value 0.0365) of observation. Bearing these patterns in mind is important when making clinical decisions; in our institution we do not consider a patient as a “non-responder” before they have been on regular a-RCE for 2 years.

Of the 10 non-responding patients, two continued on the programme as they have other significant indications (one SSP and one recurrent LU), while three have discontinued treatment. Five patients have continued despite no evidence of response in terms of hospital attendance. Understandably, they feel better while on transfusions and they are very reluctant to discontinue treatment.

In our experience, regular a-RCE can be very effective in improving the clinical course of patients suffering of recurrent painful crises, as we see a greater than 75% response rate. However, as a-RCE is an expensive intervention and any service’s capacity is finite, while the risks of transfusion are well known, it is important that before entering patients on regular a-RCE for management of RPC, specific and measurable long-term targets are discussed and agreed to avoid—as much as possible—future disappointment or confrontation. Equally, given the slow and gradual improvement we observe, patients need to be counselled that treatment may take a substantial amount of time before they notice any improvement.

### 3.5. Secondary Stroke Prevention

A total of 14 patients have been on the programme for SSP. There were no patients treated for primary stroke prevention. This is explained by the fact that we only treat adult patients. During the studied period there were no patients transitioning to adult care that were high risk for stroke and on previous regular transfusions. The mean pre-transfusion Hb S level for this group was 37% (range: 26 to 48%). It should be noted that the suboptimal Hb S level achieved for some of these patients was largely the result of them choosing to have a less “intense” transfusion programme, despite advice and warning of the potential risks.

Two of these patients were looked after at a neighbouring institution and have only been with us for 1 year, during which we have facilitated their transfusions, while their original service was setting up their apheresis service. Neither of these two patients had any new neurological events in this period.

The remaining 12 have been on a-RCE for a median of 6.5 years (3.5 to 9.5 years). One patient suffered a haemorrhagic stroke, despite already being on a-RCE for RPC and SCN. A total of 2 years after the first event, he suffered a second haemorrhagic stroke. Now, 3 years on, he remains on a-RCE with no evidence of progressive vasculopathy on annual brain MRI/MRA.

A total of two patients have died. One of these patients died due to COVID-19 infection and the second died at home and at the time of writing this the post-mortem results are not yet available.

With the exception of the aforementioned patient, no one else had any further neurological complications. A total of 10 out of the 12 patients have had annual surveillance brain MRI/MRA with no evidence of progression of vasculopathy.

### 3.6. Leg Ulcers

We have treated 12 patients with recurrent leg ulcers (LU) including one patient from another institution who has been having transfusions at our service for 1 year.

Two of our patients had longstanding very extensive bilateral leg ulcers before commencing a-RCE. Over the years (4 and 5 years, respectively), we have seen great improvement especially for one of them with granulation of large areas. However, their ulcers never healed completely and are still open. One more patient with far less severe involvement experienced recurrences twice while on the program.

The remaining eight patients achieved complete remission with no recurrence of LU while on a-RCE, with the exception of one patient who had a brief recurrence provoked by a minor injury that resolved quickly. It should be noted, that three of these patients had recurrences during times they decided to discontinue transfusions, but achieved complete resolution upon restarting. All three patients restarted a-RCE in less than 6 months from discontinuing.

### 3.7. Pulmonary Hypertension/Raised TRVmax

Four patients with RHC confirmed pulmonary hypertension (PH) and an additional five patients with suggestive symptoms and signs (difficulty in breathing, hypoxaemia, raised jugular venous pressure) and TRVmax ≥ 2.8 m/s but no confirmation by RHC (PH?), have been treated with regular a-RCE for a median of 4.5 years (6–93 months). Patients from both groups experienced an immediate improvement in their symptoms and echocardiographic parameters, even after the first a-RCE procedure, as we have previously reported [12,13]. All of these patients also receive a follow up check in a pulmonary hypertension specialist clinic and are on appropriate agents such as sildenafil, anticoagulants or diuretics.

Two patients have since died: one patient with PH 6 months after commencing a-RCE secondary to biliary sepsis and one patient with PH? 4 years after starting on the program, due to end stage renal failure. Both had experienced significant improvements in their symptoms and echocardiographic parameters.

Two cases clearly highlight the immediate and dramatic beneficial effect of a-RCE, as well as the dependence on it to maintain the benefit. The first patient with PH we ever treated and who experienced a dramatic improvement decided to discontinue 26 months after starting. At that time, the patient was asymptomatic with no evidence of PH by echocardiography. Within two months and with the Hb S level having risen to 75%, the patient became increasingly breathless and an echocardiogram 4 months after discontinuing a-RCE showed a TRVmax of 3.6 m/s. The patient re-entered the programme 1 month later and that led to immediate resolution of symptoms, while a repeat echocardiogram at the end of the same month did not show any evidence of PH. More than 4 years on, this patient remains on treatment with minimal to no symptoms. Another patient with severe SCN and established CKD became progressively breathless over the space of few months and presented acute severe breathlessness and hypoxia (SaO_2_ 88% at room air conditions). An urgent echocardiogram showed a TRVmax of 3.5 m/s. The patient underwent a-RCE the following day which led to rapid resolution of symptoms, while a repeat echocardiogram the following day (48 h after the first) showed the TRVmax to have reduced to 2.5 m/s. A similar pattern has been observed for all patients. The latest patient with PH? that has entered onto the programme started with a TRVmax of 3.1 m/s and the latest echocardiogram 10 months after starting shows no evidence of PH while the patient is essentially asymptomatic.

The beneficial role of a-RCE is highlighted by the fact that two patients with confirmed PH were already on maximal medical treatment but remained significantly symptomatic until commencing on the program, while one experienced significant recurrence of her symptoms with deterioration of her TRVmax when she discontinued a-RCE, despite remaining on medical treatment. In addition, all patients with PH? experienced the same symptomatic improvement, despite not being on medical treatment.

PH has an incidence of ~10% with significant associated morbidity and mortality [14]. In addition, a TRVmax of 2.5 m/s or higher has been shown to be an independent risk factor for increased mortality [15]. Disease modifying strategies in the form of hydroxyurea or chronic transfusions along with standard PH management are recommended [16]. Given the excellent results we see in our population, a-RCE is the first line treatment for patients with confirmed PH or those who are symptomatic with elevated TRVmax in our institution.

### 3.8. Effect on Renal Function

**Figure 5:**
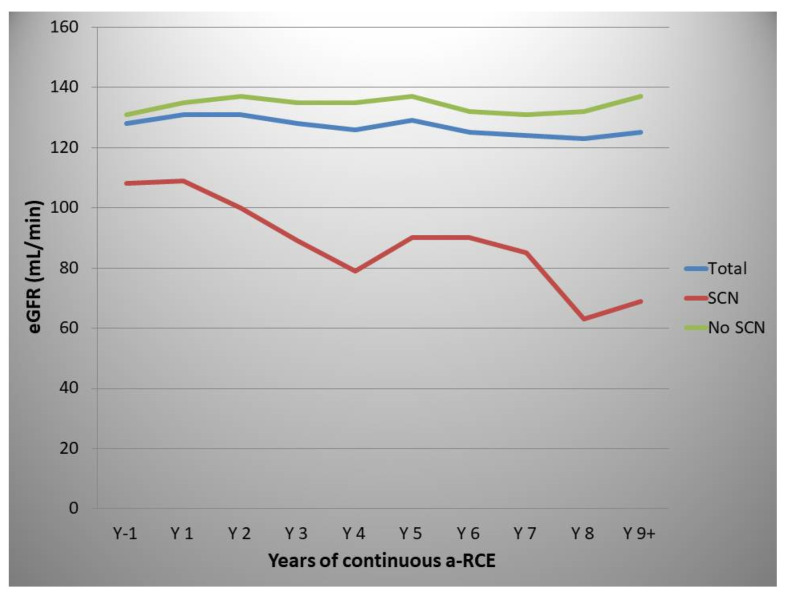
Effect of automated red cell exchange on estimated glomerular filtration rate (mL/min). Total (blue line), patients with no sickle cell nephropathy (green line), patients with sickle cell nephropathy (redline).

The mean eGFR for the whole population, one year prior to starting a-RCE, was 128 mL/min, while the relevant figure at 9+ years of a-RCE was 125 mL/min. However, there were significant differences between patients with and without SCN before commencing a-RCE. The mean eGFR 1 year prior to commencing a-RCE was 108 mL/min and 131 mL/min for patients with and without SCN, respectively (Figure 5), while at 9+ years this figure was 69 mL/min and 137 mm/min, respectively. These changes were not statistically significant, with *p* values of 0.980 and 0.8626, respectively (Kruskal–Wallis test, Graphpad Prism v 9).

A gradual decline in eGFR of 2 mL/min/year has been described for patients with sickle cell disease [4]. Our patient sample is too small and heterogeneous to draw any conclusions on any beneficial effect of a-RCE on these patients’ renal function. However, our data may suggest a possible protective effect and preservation of eGFR for patients with no evidence of SCN entering a regular a-RCE programme, although our data did not reach statistical significance.

### 3.9. Opthalmologic Complications

#### 3.9.1. Retinal Artery Occlusion 

We treated one patient who suffered bilateral retinal artery occlusion in the context of pneumococcal sepsis, accompanied by complete visual loss. Emergency a-RCE led to significant improvement but not resolution. The patient remained on a regular programme and with a continuous improvement in vision. However, the patient’s vision was not back to normal 8 months after the original event. Another patient with acute visual loss in the left eye due to acute retinal artery occlusion, underwent emergency a-RCE leading to complete resolution without any further intervention.

#### 3.9.2. Recurrent Retinal Haemorrhages

A patient suffering from recurrent left vitreous haemorrhages over a period of 4 years, who continued to experience bleeds as often as three times every week, despite repeated surgical interventions as well as a trial of hydroxycarbamide, started regular a-RCE in an attempt to improve the situation. She experienced no further bleeds after the first procedure and for the following 9 months at which point the patient decided to discontinue with a view to restart if the problem recurred.

### 3.10. Priapism

We previously reported an excellent response of cases with severe and drug resistant SP to a-RCE [17]. This involved five patients, four of whom were on the programme for a different primary indication (three SSP and one LU), while one patient was started specifically to manage his SP. All patients reported immediate cessation of such episodes upon commencing a-RCE. Their symptoms tended to recur between cycles, resolving immediately after the next procedure. This effect was gradually diminished as they stabilised on the programme and maintained consistently low Hb S levels. These patients continue to experience the same benefit with a median follow up of 6 years. Since then, we have treated two more patients with the sole indication being severe SP. One of these patients achieved immediate complete resolution, similarly to in the patients we previously observed. However, the second did not respond. Despite achieving pre-transfusion Hb S levels between 25 and 30%, his symptoms persisted with minimal, if any, improvement.

### 3.11. Other

Two patients with a history of ACS currently remain free of recurrences at 2 and 5 years of follow-up, respectively. There was a minimal improvement for the two patients on a-RCE for sickle cell hepatopathy (SCH); one progressed to overt liver failure and received a transplant. Despite remaining on the programme post-transplant and achieving pre-transfusion Hb S levels of no more than 10%, this patient lost function of the graft and succumbed 1 year later.

### 3.12. Adverse Events

Largely, these fell into three categories. (1) Line-related: A number of patients experienced bleeding from the exit site of the femoral Vas-Cath upon discharge. These episodes were generally self-limiting, with only two patients requiring a visit to the hospital, while no one required red cell transfusion. There was also one line-related deep vein thrombosis, one episode of pelvic haematoma formation, one episode of sepsis (*E. coli*) and retention of the line guide wire in one patient. Line-related problems have largely diminished over the years as peripheral access has been increasingly used. (2) Vasovagal episodes during the procedure: These are common and are associated with hypotension, sweating and a sense of dysphoria most often experienced towards the end of the procedure. Reducing the rate of the anticoagulant infusion and sometimes giving fluids pre-emptively is usually sufficient at preventing these episodes from recurring. It should be noted that no such episode was associated with hypocalcaemia due to citrate toxicity. (3) Post a-RCE fatigue: This is probably the most commonly reported side effect of the procedure. Patients usually complain of tiredness, lethargy and a non-specific sense of feeling unwell lasting usually 1 day post procedure, however, this can last for several days. None of these episodes were associated with hypocalcemia. As we can achieve post procedure Hb S levels of less than 10%, this is likely to be the result of replacing Hb S with Hb A, which releases oxygen less readily while still maintaining a relatively low haematocrit. We are currently in the early stages of commencing a study to assess the effects of a-RCE on the erythropoietic drive, which we suspect increases post procedure due to peripheral tissue oxygen depletion.

A target Hct of ~30% is standard in most centres performing the procedure in order to avoid hyperviscosity related complications. This is a valid concern. Even though the majority of red cells are HbA, a considerable proportion of Hb S red cells remain in the circulation, while other factors, such as chronic endothelial changes and alterations to adhesion molecules, must also be takin into consideration. That being said, evidence from recent studies resulting in significant increases in the patient’s Hb levels have not shown any evidence of such complications [18]. A multicentre study assessing the gradual increase in the target Hct for patients undergoing regular a-RCE may be a reasonable next step.

## 4. Closing Comments

In our experience, a-RCE is a safe intervention with very low alloimmunisation rates and no risk of iron loading. We have seen some very good responses from patients with recurrent painful crises, but it is clear that these responses build over time. We have also seen very good clinical responses from most patients with leg ulcers and stuttering priapism, in addition to excellent responses from patients with pulmonary hypertension.

We consider the provision of a-RCE an essential component of any specialist service looking after patients with sickle cell disease. Setting up an apheresis service requires investment in equipment and appropriate staff. However, a-RCE is a cost-effective intervention as it reduces the need for iron chelation drugs [2], while also reducing the number of episodes of emergency hospital attendance [19]. 

Regular monitoring and documentation of indications, targets, adverse events and responses is required at a multidisciplinary level. Additionally, careful planning is essential when attempting to match capacity to demand. Our policy is to run the service at 80–85% capacity to allow for staff absences, equipment maintenance, emergency procedures and the ability to enter new patients without delay when there is an urgent clinical need.

Special consideration is required for developing a robust vascular access system, which may involve training specialist nurses (as we do), or alternatively, having clear links with other services, such as anaesthetics or interventional radiology.

An additional benefit of having an apheresis service is that by using the same equipment and skills, therapeutic plasma exchange (TPE) can also be delivered. As the plasma of patients with SCD contains a whole host of potentially harmful substances, such as proinflammatory cytokines, free haemoglobin and prothrombotic molecules, TPE may have an important role to play in the management of the acute or chronic complications of such patients [20,21,22,23].

Multicentre coordinated studies investigating improving the delivery of a-RCE, choosing haematological targets, and assessing clinical responses represent the desired next steps for the near future.

## Figures and Tables

**Table 1:** Haematological parameters before and after automated red cell exchange.

	Mean Hct (Range)	Mean % Hb S/S&C (Range)	Mean PLT × 10^9^/L (Range)
Pre-aRCE	26 (19–32)	44 (17–98)	357 (74–1037)
Post-aRCE	30 (22–37)	12 (2–63)	140 (41–575)

aRCE = automated red cell exchange, Hct = haematocrit, PLT = platelets.

## Data Availability

Anonymed data available on request.

## Supplementary Materials

The following are available online at https://www.mdpi.com/2077-0383/10/4/767/s1, Supplemental File 1: Transfusion protocol template.
