# Supplementary material for: Automated Red Cell Exchange in the Management of Sickle Cell Disease
Source: J Clin Med. 2021 Feb 15;10(4):767. doi: 10.3390/jcm10040767 (PMC7918980; doi:10.3390/jcm10040767)
Supplement: Supplementary file 1 [file jcm-10-00767-s001.pdf]

HOMERTON HOSPITAL NHS TRUST – HAEMATOLOGY DEPARTMENT
BLOOD TRANSFUSION PROGRAM

NAME:

INDICATION:

HOSPITAL NO:

DOB:
REQUIREMENTS: **Automated RBC exchange transfusion via Spectra Optia**
X units RBC
Every x weeks
Target: Hct x% & Hb S <10%

RED CELL GENOTYPE

PROTOCOL:
Pre-Tx: FBC + retics + Hb S% + ferritin
U & e + lft + Ca/Mg + coagulation screen
Post-Tx: FBC + retics + Hb S%
U & E + LFT + Ca/Mg + coagulation screen

<u>Discus post-transfusion results with Haematology Consultnat before removing IV access if</u>

a. the post-transfusion haematocrit is more than one unit from the target haematocrit and / or
b. the platelet count is $\leq$ 50 x10^9/L

START DATE:

IRON CHELATION:

**Year**	**Mean serum ferritin ug/l**	**Hepatic iron Ferriscan mg/grm dry weight**

Mean pre-ARCET HbS:
Antibody Screen:
HIV/HBV/HCV:

<u>Patient consented for transfusion in the outpatient clinic.</u>

Last MDT Review: xx/xx/xxxx

Updated: xx/xx/xxxx
(Clinician in charge)
